# Moyamoya Disease in a Young Female With Neurofibromatosis Type 1

**DOI:** 10.7759/cureus.19121

**Published:** 2021-10-29

**Authors:** Yusuf Mehkri, Lorena Figueredo Rivas, Rebecca Jules, Ibrahim S Tuna, Brian L Hoh, Hans H Shuhaiber

**Affiliations:** 1 Department of Neurosurgery, University of Florida College of Medicine, Gainesville, USA; 2 Department of Neurology, University of Florida College of Medicine, Gainesville, USA; 3 Department of Radiology, University of Florida College of Medicine, Gainesville, USA

**Keywords:** moyamoya disease, neurofibromatosis type 1 (nf-1), ischemic stroke, aspirin, revascularization

## Abstract

Moyamoya disease (MMD) is a rare cerebrovascular disease characterized by progressive stenosis of the terminal portions of the internal carotid arteries (ICAs) and the development of a network of abnormal collateral vessels. This case depicts a 25-year-old African American female patient with neurofibromatosis type 1 (NF-1), whose initial hospital presentation occurred in a hypertensive emergency setting. Surveillance studies with magnetic resonance imaging (MRI) revealed multiple asymptomatic right cortical strokes. Genetic testing evidenced a novel, unique pathogenic variant on the NF-1 gene*. *The patient underwent combined bypass surgery first and then was placed on aspirin and a blood pressure control regimen. Our case illustrates the need for clinicians to include moyamoya disease in the list of differential diagnoses when encountering a young patient, without major risk factors, presenting with ischemic stroke. It should be considered even with no known history of previously diagnosed MMD or NF-1, as these pathologies may have yet to be evaluated in subclinical cases.

## Introduction

Moyamoya disease (MMD), a rare cerebrovascular disease characterized by progressive stenosis of the internal carotid arteries (ICAs) and the development of abnormal collateral vessels, was first described in 1957 in Japan and is thought to have a predilection for people of Asian descent [1,2]. The highest rates of MDD were found in Japan with a prevalence of 3.16-10.5/100,000 and an incidence of 0.35-1.13/100,000/year [1,2]. On the other hand, it is a fairly rare occurrence in the African American community, with a reported incidence of 0.13/100,000 [3]. Nonetheless, MMD is globally recognized as one of the major causes of childhood stroke.

Age of onset of MMD follows a bimodal distribution, with first onset between five and 10 years and second onset between 30 and 50 years [4]. When MMD is associated with an underlying condition such as neurofibromatosis type 1 (NF-1), it is referred to as moyamoya syndrome (MMS) [5]. Regardless of the cause, moyamoya angiopathy increases the risk of ischemic and hemorrhagic events. The specific treatment to prevent such complications is usually revascularization surgery in conjunction with therapeutics relating to the underlying disease. The exact pathophysiology of moyamoya is unknown. However, genetic factors appear to play a major role in this disorder. We present a case highlighting the clinical presentation and treatment plan for a patient with a novel NF-1 mutation who was later diagnosed with MMS.

## Case presentation

**Figure 1:**
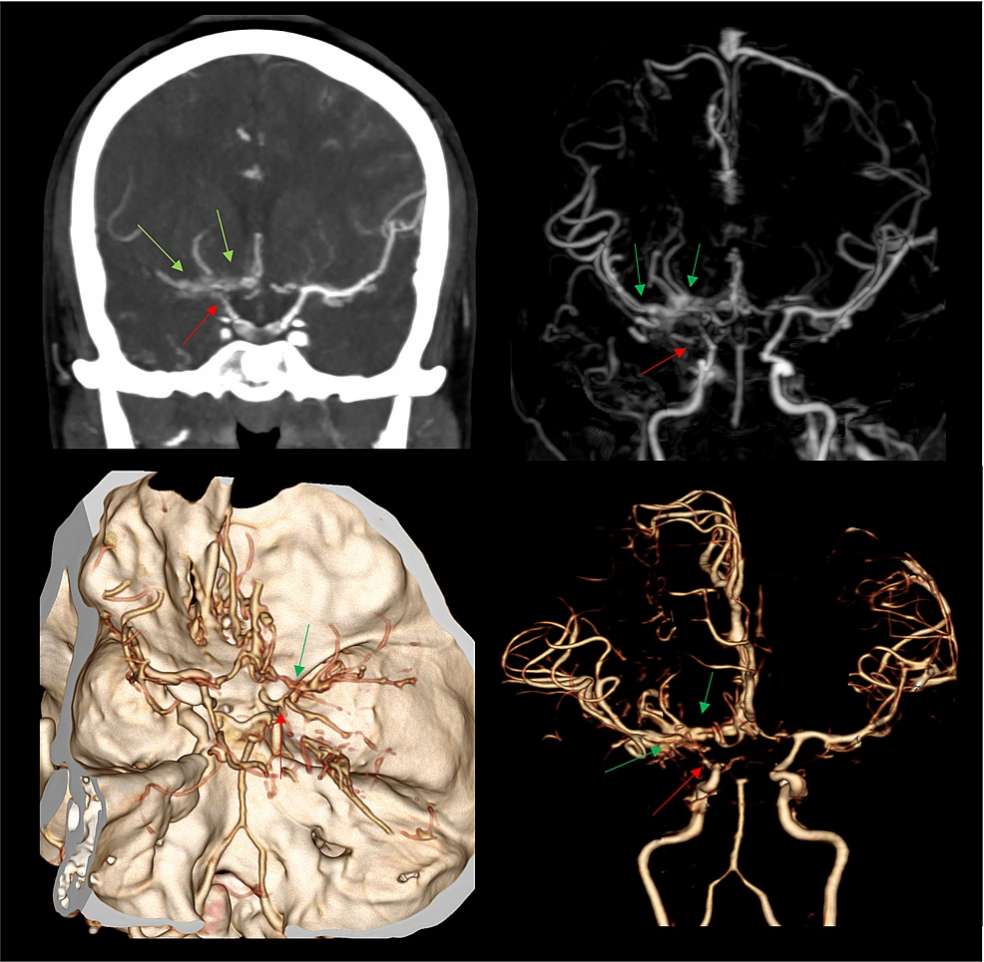
Classic complex collateralization on CTA images CTA demonstrating tapering and complete occlusion of right carotid terminus (red arrow) with multiple collateral arteries along the right carotid terminus, A1 and M1 segments (green arrows) compatible with MMD. CTA: computed tomography angiography; MMD: moyamoya disease

A 25-year-old patient with a history of hypertension and NF-1 presented with hypertensive emergency, complaining of severe headache in the setting of an unremarkable neurological examination. Computed tomography angiography (CTA) showed bilateral ICA stenosis and near occlusion of the superior division of the right M2 segment, resulting in complex collaterals and anastomotic supplies (Figure 1). Contribution from the posterior circulation via leptomeningeal collaterals was also evident. These findings were indicative of MMD; however, extracranial-intracranial (EC-IC) bypass was initially deferred due to a lack of neurovascular ischemic symptoms and the presence of auto bypass. She was instead placed on aggressive blood pressure control.

Four years later, the patient was referred to our institution for surgical evaluation given multiple, asymptomatic right cortical strokes evident on surveillance studies. Digital subtraction angiography study showed worsening MMD (right worse than left), and a small left P2-P3 aneurysm. A direct and indirect EC-IC bypass was performed, along with an encephaloduroarteriosynangiosis (EDAS). The right frontal superficial temporal branch was used for anastomosis to the M4 division of her right middle cerebral artery (MCA). The parietal branch was used for EDAS. The patient is currently at two months post-op on prophylactic aspirin and lisinopril. Genetic studies revealed a novel, unique pathogenic variant, c.1143_1144del (p.Ser382Leufs*13), found to be a premature translational stop signal in the exon 10 of the NF-1 gene that has not been previously reported in individuals with NF-1 related conditions.

Of note, the patient’s mother had a history of neurofibromas due to NF-1 and essential hypertension following her last pregnancy. She passed away from a large left basal ganglia hemorrhage with intraventricular extension secondary to her malignant hypertension and complicated by cerebral edema and significant herniation.

## Discussion

**Figure 2:**
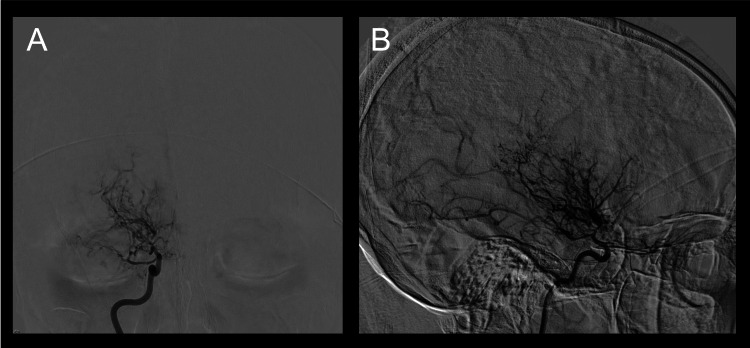
Classic puff of smoke on angiography (A) AP and (B) lateral view from catheter angiography demonstrating classic puff of smoke sign. AP: anteroposterior

MMD is typically characterized by progressive stenosis of the ICA terminals and/or the anterior and middle cerebral arteries bilaterally [6]. Rarely, the posterior circulation and the basilar artery may be involved, which was something appreciated in our young adult patient with NF-1 and MMS [7]. Reduced blood flow to the main arteries of the brain leads to the development of extensive collateral vessels that are small and irregular called “moyamoya vessels” [8]. This was also evident in our patient’s CT angiography, shown as the characteristic “puff of smoke” associated with the pathology (Figure 2).

Despite the unclear pathophysiology responsible for the vasculopathies behind MMD, associations with loci on chromosomes 3, 6, 8, and 17, as well as specific human leukocyte antigen haplotypes, have been described [9,10]. A 2008 study reported a major gene locus for autosomal dominant MMD on chromosome 17q25 [11]. The c.14576G>A variant in ring finger protein 213 (RNF213) was recently identified as a susceptibility gene variant for MMD [12-14]. The role of NF-1 in the occurrence of MMD is controversial; however, the close proximity of the NF-1 gene (17.11.2) to the familial moyamoya gene (17q25), could potentially justify the relationship between these two pathologies [15]. Genetic studies in our patient revealed a novel, unique pathogenic variant, c.1143_1144del (p.Ser382Leufs*13), found to be a premature translational stop signal in the exon 10 of the NF-1 gene that has yet to join the literature regarding individuals with NF-1 related conditions. This syndrome has been extensively described throughout the years as a result of a loss of function mutation of the neurofibromin gene, which appears to differ from our findings [15].

The clinical symptoms in MMD differ between children and adults. Adults present with transient or permanent cerebral infarction and intracranial hemorrhage, whereas children present mainly with ischemic events [1,16]. This is likely due to tearing of the moyamoya vessels as a result of the higher blood pressures typically seen in adults with MMD [1]. Our patient presented with multiple ischemic events secondary to MMD, and her diagnosis occurred at the age of 21 years.

The gold standard diagnosis for MMD is cerebral conventional angiography with ICAs, external carotid arteries, and vertebral arteries injection. It allows for the visualization of both the stenotic vessels and detailed mapping of collateral networks which is essential for treatment planning. Medical management in the form of antiplatelet therapy is indicated in all patients with MMD who have suffered from ischemic events [17]. Aspirin is often used at 50-100 mg daily in children [18]. Antiplatelet therapy may be used alone if the revascularization therapy is delayed due to recent ischemic events, infection, or if the patient is clinically asymptomatic with no evidence of markedly impaired cerebral perfusion [18]. On initial presentation, our patient had adequate spontaneous revascularization and surgery was therefore deferred.

Surgical management is the mainstay of treatment in MMD. In symptomatic patients with MMD, bypass surgery is more effective than conservative treatment to prevent future strokes. Direct bypass seems to reduce risk of future stroke more than indirect bypass. The combined approach also been associated with better outcomes than the indirect bypass [3,19]. Our patient underwent combined bypass surgery first and then was placed on aspirin. A retrospective study showed that this postoperative aspirin therapy might improve outcome based on the modified Rankin scale measurement without increasing risk of intracranial hemorrhage. However, it also found that postoperative aspirin administration did not reduce the incidence of bypass graft occlusion or the incidence of postoperative ischemic stroke [20]. More studies are needed to provide evidence for postoperative antiplatelet therapy in MMD management, especially when combined with different surgical approaches.

## Conclusions

In conclusion, we recommend that clinicians include MMD to the list of differential diagnoses when encountering a young patient presenting with ischemic stroke. It should be considered even with no known history of previously diagnosed MMD or NF-1, as these pathologies may have yet to be evaluated in subclinical cases. The management of MMD and MMS depends primarily on symptomaticity. Asymptomatic disease may be managed with aspirin with possible revascularization in the future, whereas symptomatic disease requires immediate surgical revascularization.
